# The phosphatase PPM1A controls monocyte-to-macrophage differentiation

**DOI:** 10.1038/s41598-017-18832-7

**Published:** 2018-01-17

**Authors:** Samuel R. Smith, Kaitlyn Schaaf, Nusrah Rajabalee, Frederic Wagner, Alexandra Duverger, Olaf Kutsch, Jim Sun

**Affiliations:** 1 Department of Biochemistry, Microbiology and Immunology, University of Ottawa, Ottawa, Ontario Canada; 2 Department of Medicine, University of Alabama at Birmingham, Birmingham, Alabama USA

## Abstract

Differentiation of circulating monocytes into tissue-bound or tissue-resident macrophages is a critical regulatory process affecting host defense and inflammation. However, the regulatory signaling pathways that control the differentiation of monocytes into specific and distinct functional macrophage subsets are poorly understood. Herein, we demonstrate that monocyte-to-macrophage differentiation is controlled by the Protein Phosphatase, Mg^2+^/Mn^2+^-dependent 1A (PPM1A). Genetic manipulation experiments demonstrated that overexpression of PPM1A attenuated the macrophage differentiation program, while knockdown of PPM1A expression accelerated the ability of monocytes to differentiate into macrophages. We identify imiquimod and Pam3CSK4 as two Toll-like receptor agonists that induce PPM1A expression, and show that increased expression of PPM1A at the onset of differentiation impairs cellular adherence, reduces expression of inflammatory (M1) macrophage-specific markers, and inhibits the production of inflammatory cytokines. Our findings reveal PPM1A as a negative threshold regulator of M1-type monocyte-to-macrophage differentiation, establishing it as a key phosphatase that orchestrates this program.

## Introduction

Cells of the mononuclear phagocyte system (MPS) are an integral part of the innate immune system with major roles in pathogen defense, inflammation, development, tissue integrity, and metabolic homeostasis^1–3^. The MPS is comprised of monocytes, macrophages, and dendritic cells, the numbers of which are carefully regulated^2^. Imbalances in the presence or functions of these cellular subsets have been associated with increased susceptibility to pathogens, and disease states such as fibrosis, obesity, neurodegeneration, atherosclerosis and cancer^4^.

Despite the importance of the MPS, only in the last several years has the full complexity of monocyte and macrophage subsets, the differentiation capacity of these subsets, their ability to proliferate, and the origin of macrophages from monocytes or from other common precursors been appreciated^3,5^. Monocytes are relatively short-lived and contribute to innate immunity through local recruitment to sites of need where they then differentiate into macrophages. In humans, three different monocyte subsets have been described that have different functions and half-lives^1^. Macrophages on the other hand, are incredibly diverse and heterogeneous, with the ability to constantly shift their functional state^1,4^.

The central dogma that tissue resident macrophages originate from circulating peripheral blood monocytes that migrate into tissues in response to various stimuli has been challenged by evidence that now show these macrophages as being derived from embryonic precursors as well as having the ability for self-renewal^1,5,6^. Despite these new findings, blood monocyte recruitment remains an important physiological process, in particular under conditions of inflammation, as local tissue resident macrophage numbers drastically decline due to emigration or cell death^1^. These infiltrating blood monocytes are the source of inflammatory macrophages as they undergo monocyte-to-macrophage differentiation. Therefore, the monocyte-to-macrophage differentiation process must be tightly controlled to maintain homeostasis and to enable the proper inflammatory response.

Stimuli that trigger monocyte-to-macrophage differentiation have been known for a long time^1,7^. For example, colony-stimulating factor (M-CSF) and interleukin (IL)-34 are involved in homeostatic control of monocyte/macrophage development^7,8^, while granulocyte-macrophage colony-stimulating factor (GM-CSF) is key in promoting monocyte-to-macrophage differentiation towards an inflammatory phenotype^7,9^. It has also been shown that the chemokine CXCL12 regulates monocyte-to-macrophage differentiation by downregulating expression of the transcription factor RUNX3^10^. However, it remains unknown what regulates the intracellular signaling pathways during monocyte-to-macrophage differentiation to keep this process in check.

We postulated that phosphatases would be ideal candidates as threshold regulators of macrophage differentiation as they are, different from kinases, relatively unspecific in their target selection and can target multiple downstream proteins or even pathways. As an example for phosphatases exerting broad control over cellular processes, previous reports have shown the involvement of the Protein Phosphatase 2A as a negative regulator of osteoblast and adipocyte differentiation^11,12^. In line with this, work from our group has recently demonstrated that another phosphatase, Protein Phosphatase, Mg^2+^/Mn^2+^-dependent 1A (PPM1A), in macrophages acts as a checkpoint for both the innate cellular immune response to viral infections (HIV) and to infection with the intracellular bacterial pathogen *Mycobacterium tuberculosis*^13,14^. Given this high level integrative function of PPM1A, we investigated whether PPM1A would act as a general threshold regulator in monocytes or macrophages.

Herein, we demonstrate that PPM1A controls monocyte-to-macrophage differentiation and acts as a threshold regulator to limit differentiation into classically activated (M1) macrophages in response to GM-CSF. This suggests that modulation of PPM1A expression levels could prevent excessive inflammatory conditions associated with uncontrolled macrophage differentiation. As such, this study reveals a key role for the host phosphatase PPM1A as a negative regulator of monocyte-to-macrophage differentiation, a fundamental biological process implicated in multiple human diseases.

## Results

### PPM1A is kinetically upregulated during monocyte-to-macrophage differentiation

**Figure 1:**
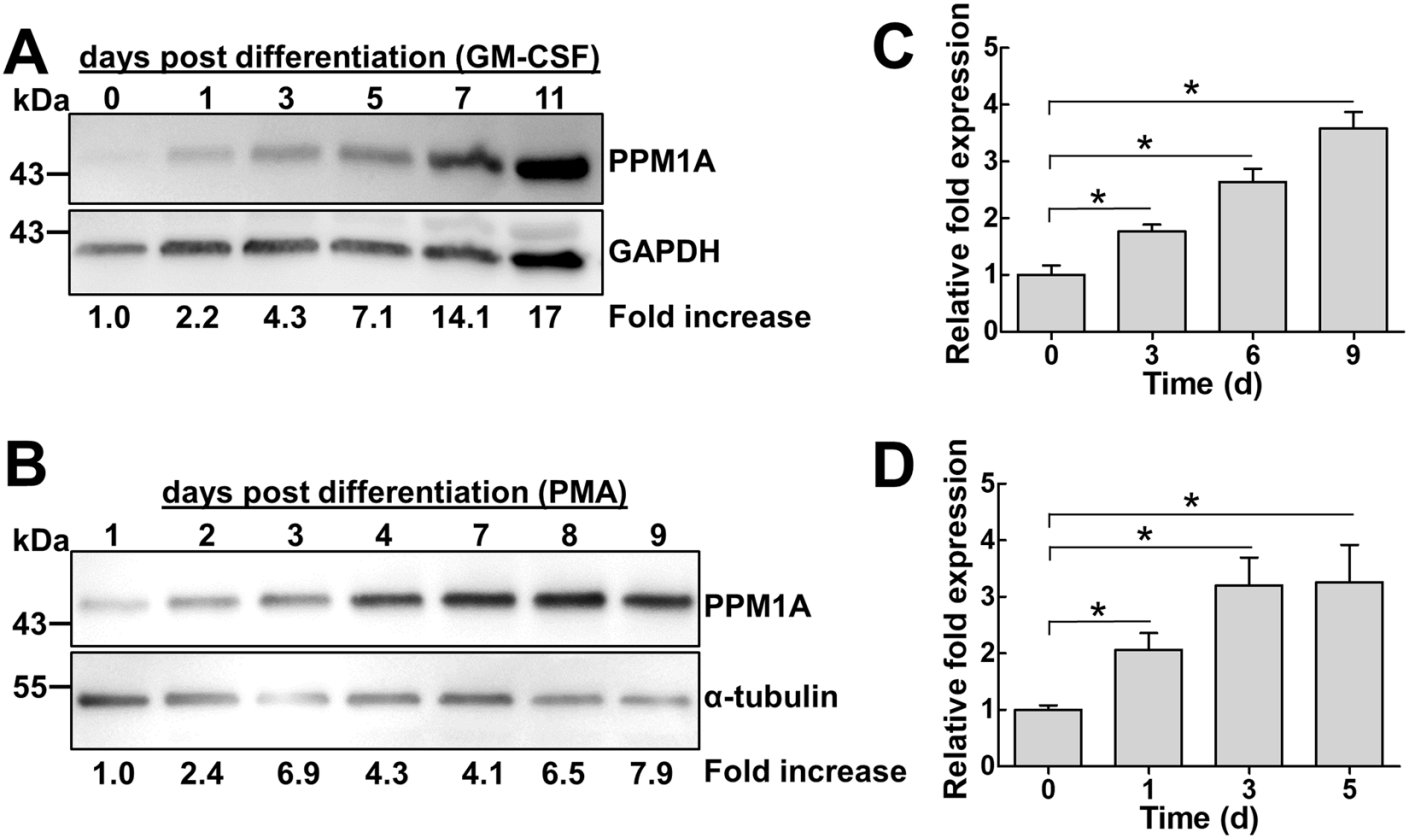
PPM1A is kinetically upregulated during monocyte-to-macrophage differentiation. (**A**) Primary human CD14^+^ monocytes or (**B**) THP-1 monocytes were stimulated with GM-CSF (5 ng/ml) or PMA (100 ng/ml) to induce macrophage differentiation, respectively. Cell lysates were prepared at the indicated days post differentiation and PPM1A protein levels were analyzed by Western blotting. Densitometry analysis was performed by ImageJ to quantify PPM1A band intensities as normalized to GAPDH or α-tubulin and fold increase of PPM1A levels are expressed relative to cells prior to differentiation. The fold increase is specific to the representative blot shown. Quantitative real-time PCR was used to measure the relative expression levels of PPM1A mRNA in (**C**) primary hMDMs at day 0, 3, 6, and 9 post GM-CSF induced differentiation or (**D**) THP-1 cells at day 0, 1, 3, 5 post PMA-induced differentiation. The ΔΔCT method was used for data analysis using GADPH as a control and the relative fold expression differences was normalized to day 0 as 1.0. Data in C-D represent the means ± S.D. of three independent experiments. *p < 0.01 relative to day 0 control cells.

Given our hypothesis that PPM1A is involved in monocyte-to-macrophage differentiation, we would expect to observe an associated increase or decrease in the expression of PPM1A during this process. To address this, we measured PPM1A protein levels as a function of differentiation time in primary human monocyte derived macrophages (hMDM). Western blot analysis showed that PPM1A was kinetically upregulated in primary hMDMs up to 17-fold by day 11 post GM-CSF-induced differentiation relative to monocytes on day 0 (Fig. 1A).

As genetic manipulations of primary monocytes/macrophages are notoriously difficult, we sought to examine the effect of genetic perturbations of PPM1A expression using THP-1 cells. THP-1 monocytes/macrophages are a suitable model as they exhibit many biological features of primary human monocytes/macrophages^15–17^, while sharing similar regulation of PPM1A expression during pathogen invasion^14^. While differentiation of primary monocytes is normally induced by stimulation with GM-CSF or M-CSF^7^, THP-1 monocytes differentiate into macrophages in response to Protein Kinase C agonists, such as Phorbol 12-myristate 13-acetate (PMA)^17,18^ or byrostatin^19^. Western blot experiments showed that PPM1A protein levels indeed increased up to 8-fold over the course of a 9-day PMA-induced differentiation period in THP-1 cells (Fig. 1B), confirming that THP-1 cells can be used to study the role of PPM1A in monocyte-to-macrophage differentiation. A key difference between primary monocytes and THP-1 monocytes is the time it takes to fully differentiate into macrophages. While THP-1 monocytes can fully differentiate into macrophages within 2–3 days, primary human monocytes need 5–10 days to fully differentiate. As such, the particular cell type used for each experiment herein determines the timing of our experiments.

To investigate whether the increase in PPM1A protein levels occurred at the transcriptional level, we performed quantitative real-time PCR (qRT-PCR) on the expression of the *PPM1A* gene. Quantitative RT-PCR showed a 1.8 to 3.6-fold increase in the mRNA expression levels of *PPM1A* in GM-CSF-differentiated primary hMDMs over a 9 day period (Fig. 1C). The same phenotype was observed in PMA-differentiated THP-1 macrophages where *PPM1A* expression levels increased to 2.1–3.3-fold over a 5 day period (Fig. 1D), confirming again that THP-1 cells are representative models for human monocytes in the context of PPM1A regulation. These data show that expression of *PPM1A* increases during monocyte-to-macrophage differentiation, linking PPM1A expression levels to the two distinct differentiation states.

### Efficiency of monocyte-to-macrophage differentiation is controlled by PPM1A expression levels

To investigate whether PPM1A plays a key role in controlling macrophage differentiation, we generated PPM1A overexpressing (THP-PPM1A) and PPM1A knockdown (THP-ΔPPM1A) THP-1 monocytes^13^. The xCELLigence Real Time Cell Analysis system^13,20^ (RTCA) was used to kinetically measure the differentiation status of THP-1 monocytes that had overexpressed or knocked-down PPM1A expression^13,14^. Using an electrode network on the bottom of a cell culture plate, the RTCA system measures cell adherence, a marker of macrophage differentiation. This is achieved by measuring changes in impedance between the electrodes, which is then translated into a Cell Index (CI) measurement, a dimensionless value. An increase in the CI reflects an increase in macrophage adherence (differentiation). To support the credibility of using adherence (measured by RTCA system) as a tool to distinguish monocytes from macrophages, we demonstrate that the CI only increases when GM-CSF is used to differentiate primary human monocytes into macrophages, but not when GM-CSF is absent (Supplementary Fig. 1A). We further show that increasing CI correlates to increased expression of macrophage specific markers including CD68, CD80 and CD86 (Supplementary Fig. 1B). Together, this supports the use of RTCA as an indicator for monocyte-to-macrophage differentiation.

**Figure 2:**
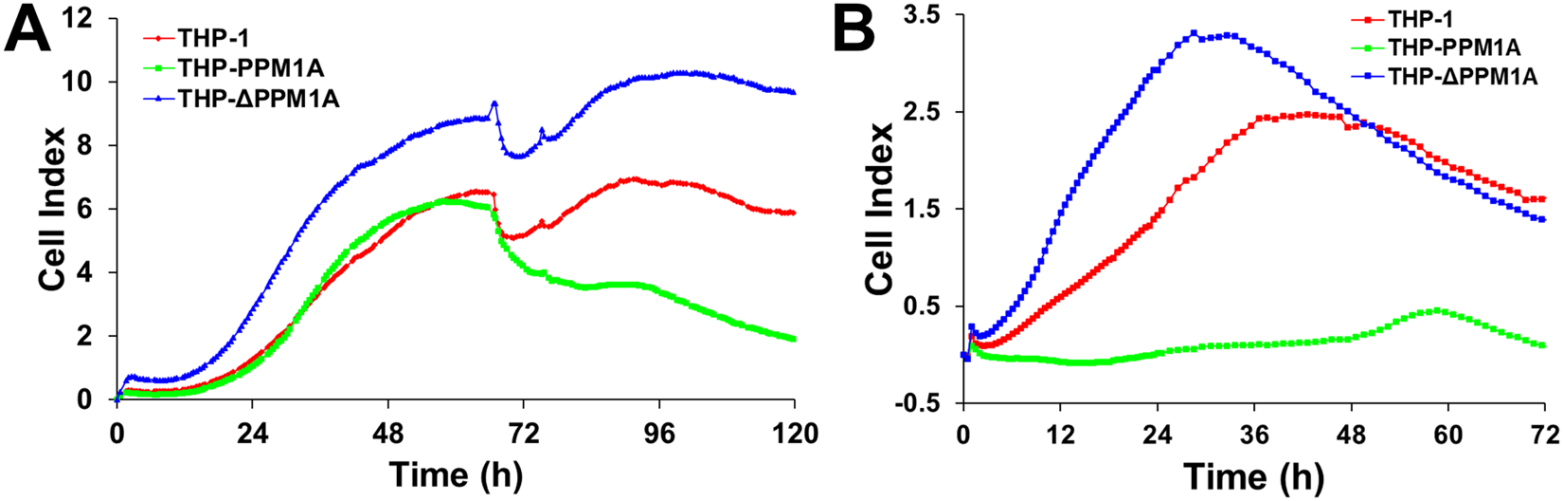
Efficiency of monocyte-to-macrophage differentiation is regulated by PPM1A. THP-1, THP-PPM1A, and THP-ΔPPM1A monocytes were treated with (**A**) PMA (100 ng/ml) or (**B**) Bryostatin (10 nM) to induce macrophage differentiation. Differentiation (adherence) was measured kinetically by the Real-time Cell Analysis (RTCA) system. The Cell Index (CI) is an arbitrary unit proportional to the strength of adherence. CI measurements were made every 30 min and represent the average of 3 independent wells.

RTCA data showed that PMA-induced differentiation of THP-ΔPPM1A was accelerated and reached higher levels of adherence compared to parental THP-1 cells (Fig. 2A). Consistent with a role for PPM1A in the control of monocyte-to-macrophage differentiation, while differentiation kinetics of THP-PPM1A cells were initially similar to parental THP-1 cells, these cells were unable to maintain a stable differentiated phenotype 72 h post differentiation as indicated by a decrease in Cell Index (Fig. 2A). This phenotype was reproduced using bryostatin as a differentiation stimulus^19,21^. Here the inhibitory effect of PPM1A was even more pronounced, as THP-PPM1A cells did not differentiate in response to bryostatin (10 nM), and were retained in a monocytic, non-adherent state (Fig. 2B). As with PMA-induced differentiation, THP-ΔPPM1A monocytes responded to bryostatin with accelerated and increased strength of adherence compared to parental THP-1 cells (Fig. 2B). These data suggest that the initial relatively low PPM1A expression levels in monocytes are essential to allow for MDM differentiation and suggest that PPM1A has a threshold function.

### Toll-like receptor agonists trigger PPM1A expression

Given the difficulties to efficiently genetically manipulate PPM1A expression in primary monocytes/macrophages, we chose instead to identify pharmacological inducers of PPM1A expression. Importantly, to examine the effect of high PPM1A expression levels on monocyte-to-macrophage differentiation, these compounds would need to induce PPM1A expression without triggering differentiation. While it is clear that stimuli such as GM-CSF and PMA trigger increased PPM1A expression over time, it is unclear whether these stimuli do so by initiation of the cellular differentiation program or directly induce PPM1A expression.

**Figure 3:**
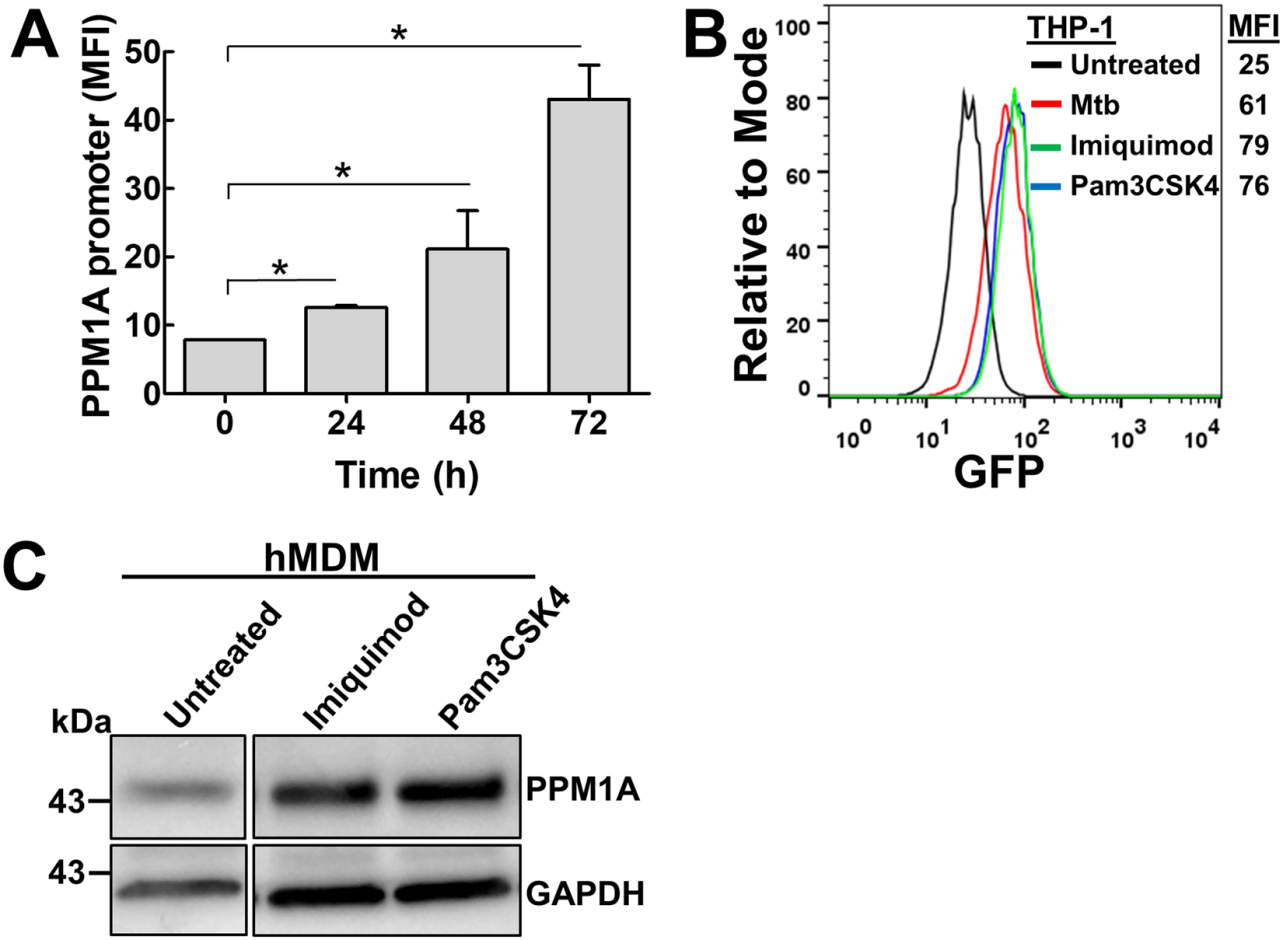
TLR agonists induce upregulation of PPM1A expression. (**A**) THP-PPM1A^prom^ cells were treated with PMA (100 ng/ml) to induce macrophage differentiation. At the indicated time points post differentiation, cells were analyzed by flow cytometry to measure the amount of GFP production as a function of PPM1A promoter activity. MFI: mean fluorescence intensity of GFP signal. Results represent the means ± S.D. of three independent experiments. *p < 0.01 relative to day 0 control cells. (**B**) PMA-differentiated THP-PPM1A^prom^ cells were left untreated, infected with Mtb, or stimulated with imiquimod (5 µg/ml) or Pam3CSK4 (300 ng/ml) for 48 h. Flow cytometric analysis was used to measure the amount of GFP production as a function of PPM1A promoter activity. MFI: mean fluorescence intensity of GFP signal. (**C**) Cell lysates from GM-CSF-differentiated hMDMs treated with imiquimod (5 µg/ml) or Pam3CSK4 (300 ng/ml) for 48 h were prepared and used to analyze PPM1A protein levels by Western blotting. The untreated lane was cropped from the same gel/blot for easier comparison to the experimental lanes as the original blot contained irrelevant lanes in between the untreated and treated samples. The full-length/uncropped blot is presented in Supplementary Fig. 3.

To identify inducers of PPM1A expression, we generated a *PPM1A* gene promoter reporter cell-line in THP-1 monocytes (THP-PPM1A^Prom^). In this reporter cell line, transcriptional activation of the PPM1A promoter would be proportional the level of GFP fluorescence. PMA-induced differentiation of THP-PPM1A^Prom^ monocytes resulted in a 5-fold increase in GFP signal over 72 h as the cells matured into macrophages (Fig. 3A). While macrophages are known to exhibit autofluorescence, this cannot account for the signals we observed with the PPM1A promoter reporter cells during differentiation given that autofluorescence of PMA-differentiated macrophages reached a maximum mean fluorescence intensity (MFI) of ~25 (Fig. 3B). Consistent with our previous report^14^, infection of PMA-differentiated THP-PPM1A^prom^ macrophages with *Mycobacterium tuberculosis* (Mtb) also induces PPM1A promoter activity in THP-PPM1A^Prom^ cells (Fig. 3B).

We performed a drug repositioning screen (~2000 compounds), and identified two Toll-like receptor (TLR) agonists that strongly induced PPM1A promoter activity: Pam3-Cys-Ser-Lys4 (Pam3CSK4), a synthetic triacylated lipopeptide that triggers TLR1/2^22^ and imiquimod, an imidazoquinoline amine analog to guanosine that triggers TLR7^23^ (Fig. 3B). As TLR agonists mimic pathogen associated molecular patterns (PAMP), the identification of imiquimod and Pam3CSK4 is consistent with our previous findings that both Mtb and HIV-1 infection directly triggered upregulation of PPM1A^14^. Western blot experiments further confirmed that imiquimod and Pam3CSK4 indeed increased the production of PPM1A protein in primary human macrophages (Fig. 3C). Monocytes treated with imiquimod or Pam3CSK4 retained >90% viability after 48 h of treatment and exhibited negligible differences in proliferation rates, thereby showing no cytotoxic effects when used at the concentration that induces PPM1A expression (Supplementary Fig. 2A,B). Importantly, these two compounds do not trigger monocyte-to-macrophage differentiation, allowing us to examine the effect of elevated PPM1A levels prior to the onset of a differentiation stimuli.

### Pharmacologic perturbation of PPM1A alters monocyte-to-macrophage differentiation

The identification of imiquimod and Pam3CSK4 as PPM1A expression inducers allowed us to test the effect of PPM1A expression on monocyte-to-macrophage differentiation.

**Figure 4:**
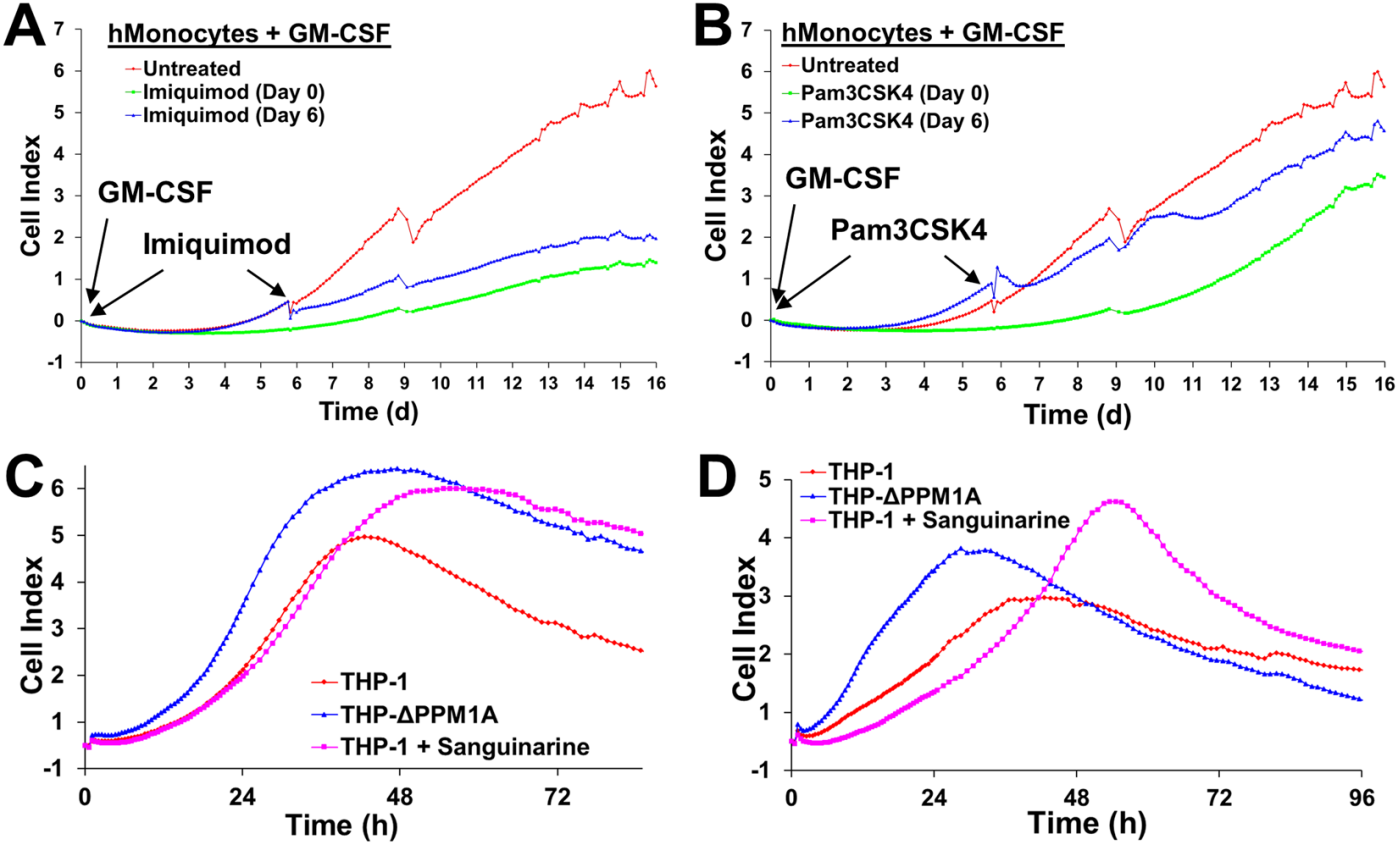
Pharmacologic perturbation of PPM1A affects monocyte-to-macrophage differentiation. Primary human monocytes (hMonocytes) were differentiated with GM-CSF (5 ng/ml) (day 0) and either left untreated or treated with (**A**) imiquimod (5 µg/ml) or (**B**) Pam3CSK4 (300 ng/ml) on day 0 or day 6. Changes in the Cell Index, proportional to the strength of differentiation/adherence, was measured kinetically over 16 days using the RTCA system. Untreated THP-1 and THP-ΔPPM1A monocytes and sanguinarine (300 nM) pre-treated THP-1 monocytes were differentiated with (**C**) PMA (30 ng/ml) or (**D**) Bryostatin (10 nM). Changes in the Cell Index was measured kinetically over 4 days using the RTCA system. CI measurements were made every 30 min and represent the average of 3 independent wells.

Similar to THP-1 cells overexpressing PPM1A (Fig. 2A), primary human monocytes treated with imiquimod or Pam3CSK4 differentiated poorly in response to GM-CSF compared to mock treated human monocytes (Fig. 4A,B). As indicated by adherence data using RTCA, addition of imiquimod on day 0 of differentiation rendered monocytes unable to differentiate into fully adherent macrophages (Fig. 4A). Imiquimod also affected the differentiation status when added on day 6, long after the actual differentiation program was initiated by GM-CSF. Treatment with Pam3CSK4 on day 0 of differentiation also drastically delayed the kinetics of differentiation (Fig. 4B). However, when Pam3CSK4 was added on day 6 post initiation of differentiation, its effect on macrophage differentiation was less pronounced (Fig. 4B). This difference could be attributable to the fact that imiquimod and Pam3CSK4 activate two distinct TLRs, which may regulate PPM1A expression differently in terms of kinetics. Collectively, these data suggest that increased PPM1A expression inhibits the efficiency of monocyte-to-macrophage differentiation.

While we identified compounds that could increase PPM1A expression, this limited drug reposition screen did not reveal any compounds that could lower its expression. However, we and others have previously demonstrated that the plant alkaloid sanguinarine could inhibit the phosphatase activity of PPM1A^13,24^. We here show that pre-treatment of THP-1 monocytes with sanguinarine to inhibit PPM1A phosphatase activity increased adherence of these cells during differentiation by PMA and bryostatin closer to levels achieved using THP-ΔPPM1A macrophages (Fig. 4C,D). While the amplitude of adherence was increased, sanguinarine treatment did not affect the kinetics of differentiation, possibly because this may require higher concentrations that are toxic to the cells.

**Figure 5:**
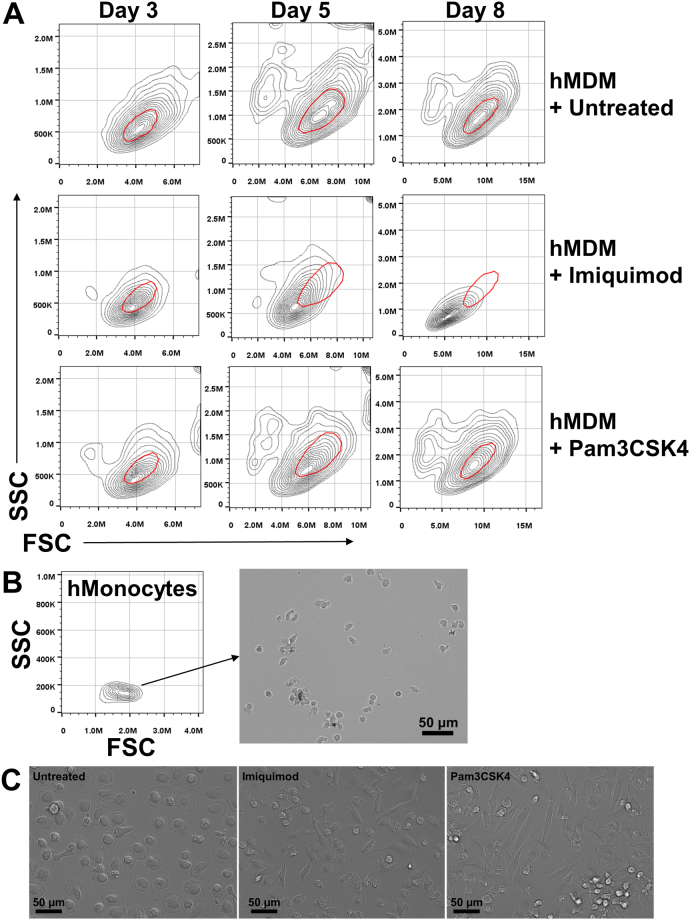
Elevated PPM1A levels alter morphology of differentiated macrophages. (**A**) Primary human monocytes were differentiated with GM-CSF (5 ng/ml) and simultaneously mock treated (untreated) or treated with imiquimod (5 µg/ml) or Pam3CSK4 (300 ng/ml). On days 3, 5, and 8, hMDM morphology was analyzed by flow cytometry. (**B**) Primary human monocytes in the absence of GM-CSF were analyzed by flow cytometry and bright-field microscopy. (**C**) Representative bright field images of macrophages treated as in (**A**) on day 5 post differentiation were used to visualize cell morphology.

Given the decreased adherence of primary human macrophages differentiated following induction of PPM1A expression, we examined whether this correlated to an altered morphology. Indeed, flow cytometric analysis of primary human monocytes undergoing monocyte-to-macrophage differentiation following GM-CSF stimulation showed that the imiquimod and Pam3CSK4 treatments reduced both the side and forward-scatter of these cells, indicative of decreased cell granularity and size, respectively (Fig. 5A). This was particularly evident in the imiquimod treated cells (Fig. 5A, middle panel). The effect of Pam3CSK4 on monocytes was moderate, but visible on day 3 and 5 post differentiation, but by day 8, showed similar flow cytometry scattering profiles as control untreated cells (Fig. 5A, lower panel). This is consistent with RTCA analysis that showed Pam3CSK4 treated monocytes produced a Cell Index that was closer to levels of control cells by later time points of differentiation compared to imiquimod treated monocytes (Fig. 4A,B).

To visualize the changes in cell morphology indicated by flow cytometry, we performed microscopy analysis. Untreated human monocytes are typically smaller in size and are not adherent to the culture wells (Fig. 5B). Strikingly, while mock treated GM-CSF differentiated macrophages resulted in the typical flattened, pancake-like shapes indicative of activated (M1) morphology^25^, monocytes differentiated in the presence of imiquimod or Pam3CSK4 transformed into elongated cell shapes, resembling alternatively activated macrophages^25^ (Fig. 5C). These data suggest that elevated PPM1A expression at the onset of a differentiation signal can predispose the differentiating monocytes into a particular phenotype. Interestingly, while differentiation stimulated by GM-CSF induced a dramatic increase in PPM1A expression (Fig. 1A), M-CSF-induced differentiation of primary human monocytes resulted in a much more modest 2-fold increase in expression of PPM1A by day 11 post differentiation (Supplementary Fig. 4). Consistent with cell morphology data, this suggests that PPM1A plays a larger role during the differentiation of macrophages that are produced by GM-CSF stimulation compared to M-CSF stimulated differentiation^7,26^.

### PPM1A expression reduces inflammatory macrophage surface markers

**Figure 6:**
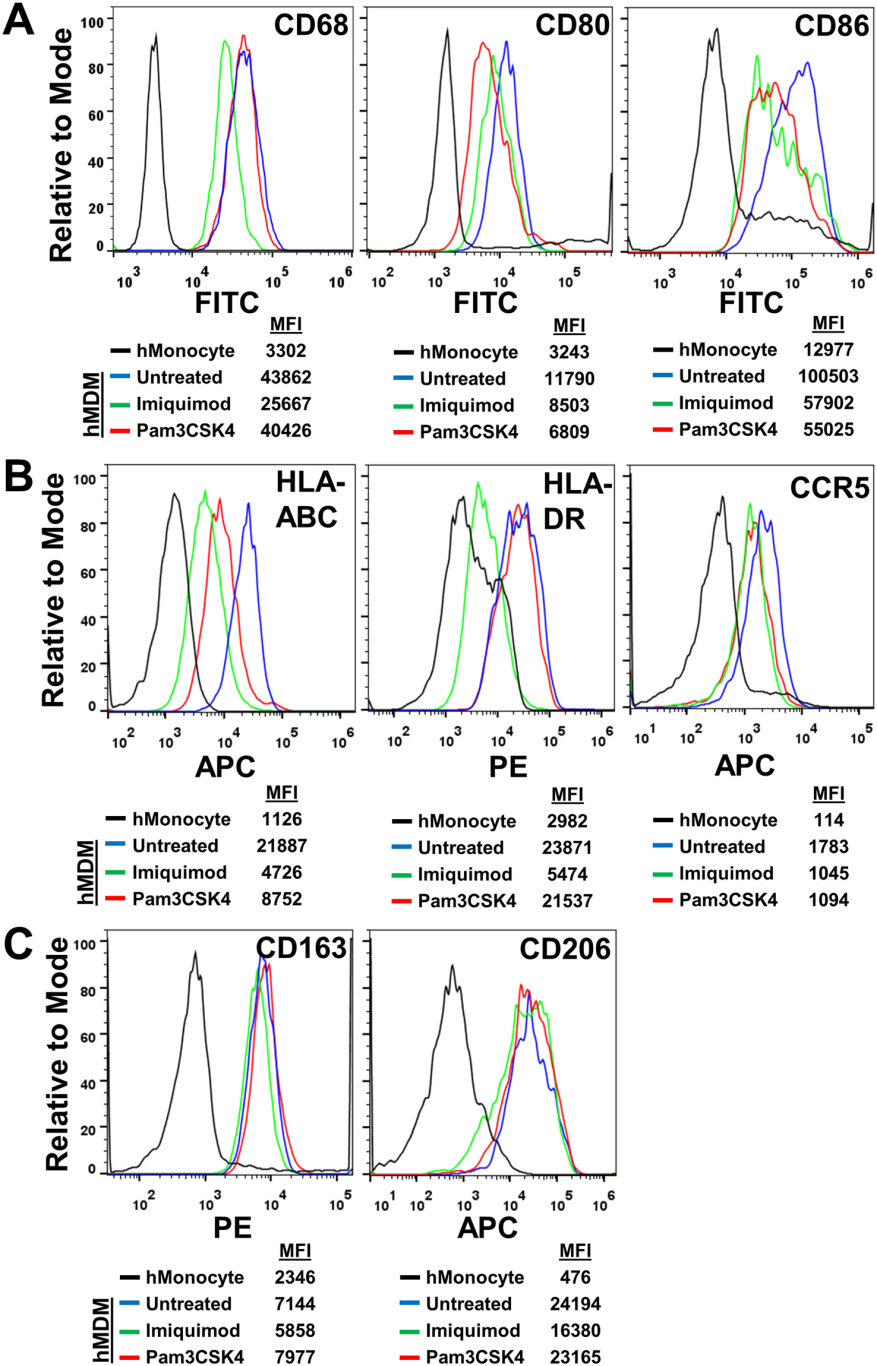
PPM1A expression reduces inflammatory macrophage surface markers. Primary human monocytes were differentiated with GM-CSF (5 ng/ml) and simultaneously mock treated (untreated) or treated with imiquimod (5 µg/ml) or Pam3CSK4 (300 ng/ml). On day 8 post differentiation, hMDMs were stained with fluorochrome conjugated specific antibody to (**A**) CD68, CD80, CD86, (**B**) HLA-ABC, HLA-DR, CCR5, and (**C**) CD163, CD206 and compared to similarly stained undifferentiated primary human monocytes (hMonocyte) from day 0. Stained cells were analyzed by flow cytometry to measure intensity of the stained macrophage markers. MFI: mean fluorescence intensity.

As our data indicated that PPM1A negatively regulates monocyte-to-macrophage differentiation, particularly towards that of an M1 morphology (Fig. 5), we would expect this to correlate with lower levels of inflammatory macrophage markers. We profiled well-characterized surface markers on primary human macrophages following GM-CSF induced differentiation in the absence or presence of imiquimod and Pam3CSK4, which served as a means to raise PPM1A levels. Staining for the classic intracellular macrophage marker CD68^27,28^, we observed a ~ 1.7-fold decrease in MFI in macrophages differentiated in the presence of imiquimod, while Pam3CSK4 treatment did not alter levels of this marker (Fig. 6A). This result is consistent with data showing that alterations in adherence and morphology is most prevalent in response to imiquimod treatment during differentiation, while Pam3CSK4 induced a smaller effect (Figs 4 and 5). While CD68 is a pan-macrophage marker and not specific for distinct subsets, CD80 and CD86 are well defined as markers for classically activated or inflammatory macrophages^26,29,30^ (M1). In this context, we show that treatment with either imiquimod or Pam3CSK4 during GM-CSF-induced differentiation for 5 days resulted in a 2-fold decrease in signal for CD86 levels compared to mock treated monocytes (Fig. 6A). In addition, both imiquimod and Pam3CSK4 treated monocytes displayed a decrease in CD80 levels compared to mock treated controls (Fig. 6A). As such, induction of PPM1A expression suppresses monocyte-to-macrophage differentiation.

In extension to these canonical macrophage-specific markers, we examined other known markers of macrophage maturation or activation, including Major Histocompatibility Complex (MHC) class I and II (HLA-A,B,C and HLA-DR) and the C-C chemokine receptor type 5 (CCR5/CD195), which have been well characterized to be upregulated upon monocyte-to-macrophage differentiation^31–33^. Expression of the MHC class I receptor HLA-ABC was reduced by ~ 4- or 2.5-fold in imiquimod or Pam3CSK4 treated monocytes, respectively on day 8 post GM-CSF induced differentiation (Fig. 6B). Conversely, levels of the MHC class I receptor HLA-DR remained unchanged in Pam3CSK4 treated cells, while that of Imiquimod treated cells were reduced by 4-fold (Fig. 6B). Levels of CCR5 were similarly reduced by treatment with either compound by ~ 1.7-fold compared to untreated controls (Fig. 6B). Together, these data further support PPM1A expression as a key negative regulator of monocyte-to-macrophage differentiation as multiple macrophage activation markers are lowered during differentiation in the presence of elevated PPM1A levels.

While CD80, CD86, MHCI/II, CCR5 are indicators for polarization towards an M1 macrophage phenotype, we examined whether PPM1A levels also affect markers of alternatively activated (anti-inflammatory) macrophages (M2). While various subsets of M2 macrophages have now been identified, it is generally accepted that CD163 (class B scavenger receptor) and CD206 (mannose receptor) indicate polarization towards an M2 phenotype^34,35^. Levels of CD163 and CD206 expression remain unchanged in macrophages differentiated following induction of PPM1A expression (Fig. 6C), suggesting that the role of PPM1A in regulation of monocyte-to-macrophage differentiation may be restricted to dampening the inflammatory phenotype, and may not affect the anti-inflammatory functions.

### PPM1A inhibits the macrophage inflammatory response

Given that overexpression of PPM1A in differentiating monocytes affects inflammatory macrophage specific markers, we postulated this would result in inhibition of the ability of these macrophage to produce an inflammatory response.

**Figure 7:**
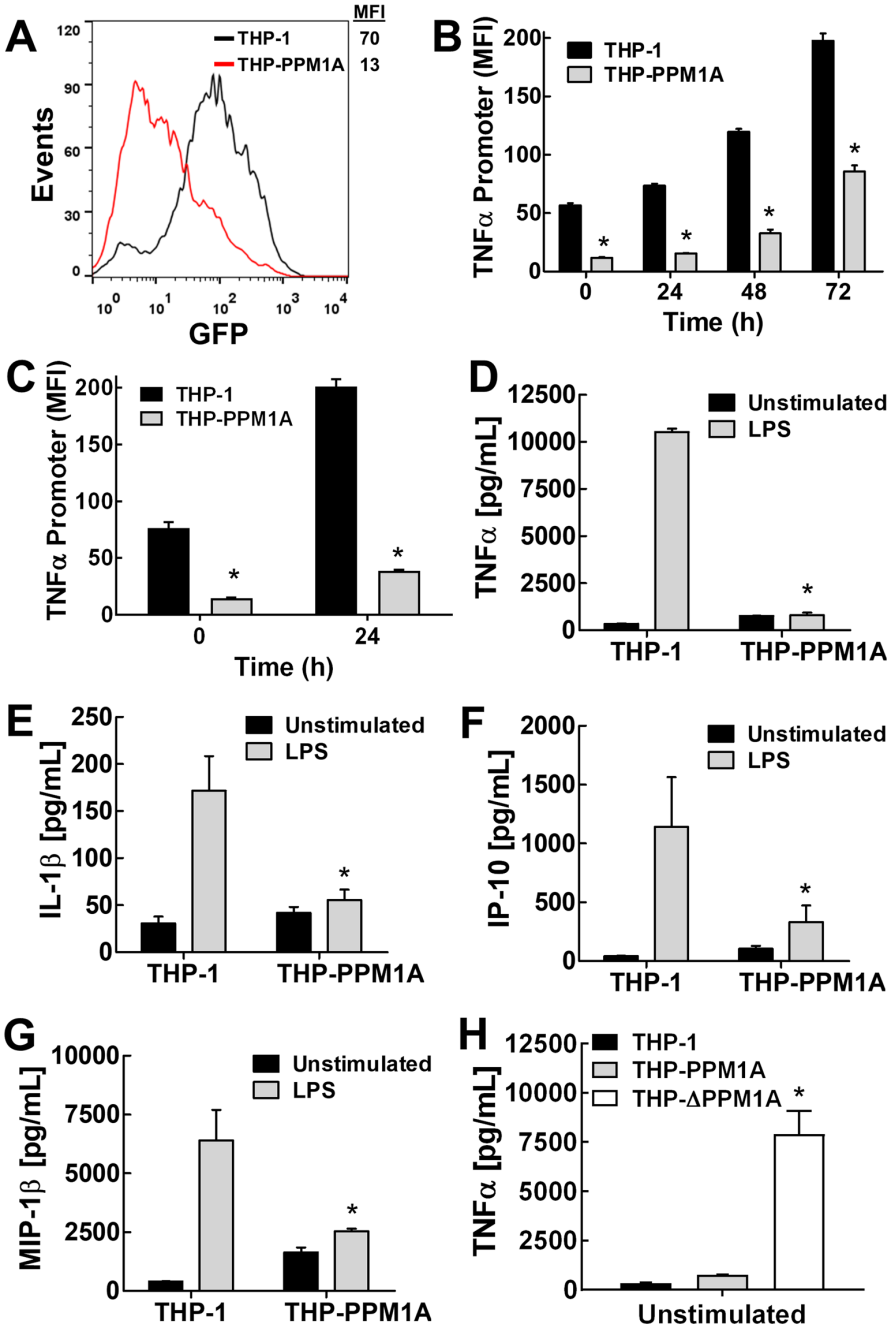
PPM1A inhibits the inflammatory response of differentiated macrophages. (**A**) Relative baseline expression of GFP in THP-TNFα^Prom^ cells compared to THP-PPM1A-TNFα^Prom^ reporter cells was measured by flow cytometry. MFI: mean fluorescence intensity of GFP signal. (**B**,**C**) THP-1 and THP-PPM1A TNFα promoter reporter monocytes were differentiated with (**B**) PMA (100 ng/ml) or stimulated with (**C**) LPS (100 ng/ml), and at the indicated days post treatment, TNFα promoter activity was measured by flow cytometry as a function of the GFP signal (MFI). (**D**–**G**) PMA-differentiated THP-1 and THP-PPM1A macrophages were mock treated or stimulated with LPS (100 ng/ml) to induce the production of cytokines/chemokines. Production of (**D**) TNFα, (**E**) IL-1β, (**F**) IP-10, (**G**) MIP-1β into the culture supernatant were measured using antibody based bead multiplex assays. (**H**) Levels of TNFα production in unstimulated PMA-Differentiated THP-1, THP-PPM1A and THP-ΔPPM1A macrophages were measured using antibody based bead assay. Data in this figure represent the means ± S.D. of three independent experiments. *p < 0.01 relative to THP-1 control cells.

We generated a THP-1 TNFα promoter driven GFP reporter cell line as a marker for the inflammatory response. Using these cells, we observed a large decrease in TNFα promoter activity when PPM1A was overexpressed (Fig. 7A). We show that while the TNFα promoter was still functional in THP-PPM1A macrophages, as indicated by the increase in promoter activity following PMA-induced differentiation, the overall magnitude of TNFα promoter activity was much lower compared to parental cells (Fig. 7B). Similarly, using lipopolysaccharide (LPS), a bacterial cell-wall lipid that induces an inflammatory response that results in increased TNFα production, we show that overexpression of PPM1A markedly inhibited TNFα promoter activity (Fig. 7C).

Consistent with these results, inflammatory cytokine and chemokine production were reduced in THP-PPM1A macrophages compared to parental THP-1 macrophages in response to LPS stimulation (Fig. 7D–G). Specifically, TNFα production was 10-fold lower in THP-PPM1A macrophages stimulated with LPS compared to THP-1 macrophages (Fig. 7D), consistent with data using the TNFα promoter cell line (Fig. 7C). In addition, we observed in THP-PPM1A macrophages about a 3-fold reduction in the production of IL-1β (Fig. 7E), a key inflammatory cytokine produced by activated macrophages that is involved in auto-inflammatory diseases^36^. The production of two key chemokines, IP-10 and MIP-1β, were also reduced in THP-PPM1A macrophages by about 3-fold relative to parental THP-1 macrophages (Fig. 7F,G). These cytokines function as chemoattractants for monocytes and NK cells to participate in establishing a pro-inflammatory environment^37,38^. Furthermore, knockdown of PPM1A in THP-1 monocytes markedly increased the production of TNFα by these cells following PMA-induced differentiation (Fig. 7H), suggesting that PPM1A is required to control the inflammatory response. Using primary human monocytes treated with Pam3CSK4 to induce PPM1A expression at the onset of differentiation, we show that these cells actually produced more IL-10, an anti-inflammatory cytokine (Supplementary Fig. 5). This further supports the role of PPM1A in shifting differentiating monocytes into an anti-inflammatory phenotype.

### Overexpression of PPM1A inhibits Akt signaling

**Figure 8:**
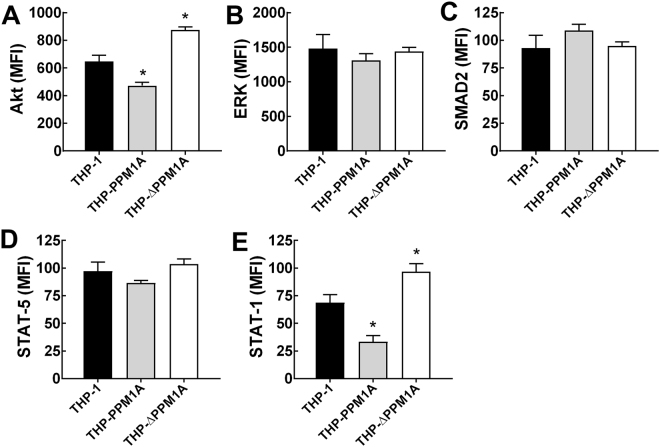
Overexpression of PPM1A inhibits Akt and STAT-1 signaling. THP-1, THP-PPM1A and THP-ΔPPM1A monocytes were differentiated with PMA for 24 h. Then, cell lysates were extracted to measure activation (phosphorylation) levels of (**A**) Akt (S473), (**B**) ERK (T185/Y187), (**C**) SMAD2 (S465/S467), (**D**) STAT-5 (Y694/699), and (**E**) STAT-1 (Y701) using antibody based magnetic bead assays (Milliplex). MFI: mean fluorescence intensity of analyte signal. Data in this figure represent the means ± S.D. of three independent experiments. *p < 0.01 relative to THP-1 control cells.

To provide more insight to how PPM1A controls differentiating monocytes, we explored activation of several potential target signaling molecules of PPM1A. Using a magnetic bead multiplex assay (Milliplex) panel for TGFβ signaling pathway molecules that PPM1A has been reported to target^39^, we show that PPM1A overexpressing THP-1 monocytes suppress Akt phosphorylation (S473), while knockdown of PPM1A in these monocytes increase Akt phosphorylation (Fig. 8A). In contrast, activation of ERK (T185/Y187) and SMAD2 (S465/S467) were unaltered by PPM1A expression (Fig. 8B,C). This result is consistent with reports that Akt activation is induced during monocyte differentiation and is essential for the survival of differentiated macrophages^40,41^. Given that STAT activation plays a major role in myeloid cell differentiation^42–44^, in particular as STAT5 phosphorylation was shown to be induced following GM-CSF stimulation^44^, we assessed whether PPM1A expression modulated the STAT signaling pathways. Surprisingly, we found that while STAT5 phosphorylation remained unchanged (Fig. 8D), STAT1 activation decreased when PPM1A was overexpressed (Fig. 8E). Consistent with this, STAT1 activation was increased when PPM1A expression was knocked down (Fig. 8E). These data suggest the possibility of a STAT1 and Akt mediated signaling pathway controlled by PPM1A that affects monocyte-to-macrophage differentiation.

## Discussion

Monocyte-to-macrophage differentiation is a fundamental biological process with important implications in many human diseases including fibrosis, cancer, neurodegeneration, and autoimmunity. As such, it is absolutely critical that this process is properly regulated to maintain the balance of various monocyte and macrophage populations as well as their functional phenotypes.

We demonstrate that the phosphatase PPM1A plays a key role in controlling monocyte-to-macrophage differentiation. Increased expression of PPM1A prior to initiation of differentiation by relevant stimuli impairs the ability of monocytes to differentiate into macrophages. We have shown that upregulation of PPM1A expression by genetic manipulation or pharmacological perturbation renders monocytes unable to differentiate into classically activated M1 macrophages using GM-CSF^26,34^, as indicated by decreased adherence, altered morphology, lower display of inflammatory macrophage-specific markers, and decreased production of inflammatory cytokines. In fact, monocytes differentiated following induction of PPM1A expression resulted in elongated cell morphology characteristic of alternatively activated macrophages^25^.

We have previously reported that both *M. tuberculosis* and HIV-1 infection of macrophages induces increased PPM1A expression^14^. As PPM1A levels are clearly upregulated during pathogen invasion, it is possible that this would translate into a systemic effect as bacterial and viral products enter circulation. Consistent with the fact that TLR agonists trigger PPM1A expression (Fig. 3), these bacterial and viral products would also affect expression of PPM1A in non-infected cells. As such, in a chronic infection scenario by pathogens such as *M. tuberculosis* or HIV-1, circulating monocytes may fail to differentiate into M1 inflammatory macrophages capable of eliciting a potent antibacterial response. In these situations, it would be beneficial to downregulate expression of PPM1A to empower the differentiation of M1-type pro-inflammatory macrophages that could eliminate the persistent bacteria or virus. Indeed, drugs that induce macrophage differentiation and activation are currently being explored as host-directed therapy to treat tuberculosis^45,46^.

Conversely, excessive macrophage differentiation contributes to several diseases involving a hyper-inflammatory response. In this scenario, pharmacological means to upregulate expression of phosphatases like PPM1A would allow for the control of inflammatory cytokine production to prevent tissue damage and restore wound healing capabilities. For instance, monocyte-to-macrophage differentiation is a critical event that exacerbates atherosclerosis by promoting an inflammatory environment within the walls of blood vessels^47^. Accordingly, inhibition of monocyte-to-macrophage differentiation by metformin reduced inflammation and subsequently plaque formation^48^. This suggests that identification of drugs that would induce upregulation of PPM1A expression to inhibit monocyte-to-macrophage differentiation could be valuable to treat atherosclerosis.

The role of phosphatases have been extensively studied in various cellular functions as they are known to regulate multiple substrates and thus signaling pathways. However, in the context of macrophage biology, and to our knowledge, only one previous report has shown the involvement of phosphatases controlling intracellular pathways to regulate differentiation. In that study, it was reported that the dual specificity phosphatase DUSP5 negatively regulated macrophage differentiation in response to M-CSF through a mechanism dependent on shutdown of the MAP kinase ERK1/2^49^. Our study extends on the results from this previous report and identifies a new role for PPM1A in GM-CSF induced monocyte-to-macrophage differentiation. Importantly, while the increase in DUSP5 expression was only transient following induction of differentiation^49^, the increase in PPM1A expression was prolonged and stable (Fig. 1). This suggests that the signaling networks controlled by upregulation of PPM1A are permanently altered following differentiation, which would indicate that PPM1A is a more attractive target to address therapeutically as modulation of its expression would lead to more pronounced effects.

Our discovery for the role of PPM1A in monocyte-to-macrophage differentiation also raises the intriguing question of whether the previously identified DUSP5 regulated differentiation pathway belongs to the same signaling pathway as PPM1A or if these are two parallel redundant pathways used by the host to limit excessive inflammation. It is possible that these two signaling pathways may intersect and converge at the level of MAPK signaling as we and others have previously shown the involvement of PPM1A in inactivating the c-Jun N-terminal kinase (JNK) and p38 response^13,50^. Further experiments are necessary to elucidate the relative contributions of DUSP5 and PPM1A controlled signaling pathways in macrophage differentiation.

In summary, our study reveals a key phosphatase that functions as a threshold regulator to control monocyte-to-macrophage differentiation. PPM1A serves as a molecular checkpoint to prevent excessive M1-type macrophage polarization to thereby limit the inflammatory response. The identification of drug compounds that could modulate the expression or activity levels of PPM1A would thus be valuable for the treatment of various human diseases that are affected by a defective monocyte-to-macrophage differentiation program.

## Methods

### Cell culture, reagents, and antibodies

THP-1 monocytes (ATCC TIB-202) and primary human monocytes/macrophages were maintained in RPMI 1640 medium supplemented with 2 mM L-glutamine and 10% heat-inactivated fetal bovine serum (FBS) at 37 °C in a humidified atmosphere of 5% CO_2_. THP-PPM1A and THP-ΔPPM1A cells were generated previously^13,14^. Human PBMCs were isolated from buffy coats by the Ficoll-Paque density centrifugation method. Monocytes were enriched by positive selection using anti-CD14 mAb-coated microbeads from Miltenyi Biotec (San Diego, CA) according to manufacturer’s protocol. Primary monocytes were differentiated with either 5 ng/ml GM-CSF (R&D Systems, Minneapolis, MN) or 10 ng/ml M-CSF (StemCell Technologies, Vancouver, Canada) for 5–11 days to obtain monocyte derived macrophages (MDM). Fetal bovine serum was obtained from Life Technologies (Grand Island, NY). Phorbol ester 13-phorbol-12-myristate acetate (PMA), puromycin, bryostatin, imiquimod, and Pam3CSK4 were purchased from Sigma (St. Louis, MO). Monoclonal PPM1A antibody were purchased from Thermo Scientific (Rockford, IL). Monoclonal mouse antibodies to GAPDH and α-tubulin were purchased from Santa Cruz (Dallas, Texas) and Cell Signaling (Danvers, MA), respectively.

### Bacteria and plasmids

The *M. tuberculosis* H37Rv derived auxotroph strain mc^2^6206 was grown in Middlebrook 7H9 medium (Difco) supplemented with 0.2% glycerol, 0.02% Tyloxapol and 10% OADC (Remel). Growth media of the auxotrophic *M. tuberculosis* strain was supplemented with 24 μg/ml pantothenate and 50 μg/ml L-leucine^51^. *Escherichia coli* strain DH5α was used for plasmid propagation, and was routinely grown in Luria-Bertani broth at 37 °C. The PPM1A promoter (transcript 1) was constructed using the entire exon 1 sequence (410 bp) flanked by 1355 bp upstream of it and the first 20 bp of Exon 2, resulting in a final sequence of 1785 bp. This full promoter sequence was generated using custom gene synthesis by Genscript (Piscataway, NJ). The PPM1A promoter sequence was then cloned into the lentiviral vector pEZX-LvPF02 (Genecopoeia, Rockville, MD) directly upstream of a GFP coding sequence to generate the final PPM1A promoter reporter construct. Lentiviral TNFα promoter reporter expressing GFP was purchased from Genecopoeia (Product ID: HPRM25564).

### Generation of THP-1 reporter cells

HEK 293T cells seeded at 50% confluency were transfected with lentiviral plasmids expressing promoter-GFP reporter constructs using FuGENE (Promega, Madison, WI). Culture supernatants were harvested after 48 h and 72 h, aliquoted, and stored at −80 °C. The supernatants containing lentiviral particles were used to transduce THP-1 cells. Cells were selected by puromycin and analyzed by promoter responsiveness to relevant stimuli.

### Detection of gene expression by quantitative PCR

Total RNA was isolated from THP-1 or primary monocytes/macrophages using the Qiagen RNeasy mini kit (Qiagen). 250–500 ng of total RNA was used in the cDNA synthesis reaction using the iScript reverse transcription supermix (Bio-rad, Hercules, CA). 2 ul of synthesized cDNA (out of 20 ul reaction) was used to analyze gene expression of *PPM1A* by real-time PCR on a CFX Connect Real-Time PCR Detection System using commercial primers (PrimePCR SYBR Green assay, Bio-Rad) in combination with the SsoAdvanced universal SYBR Green supermix (Bio-Rad). Amplification parameters were 95 °C for 30 s, followed by 40 cycles of 95 °C for 15 s alternating with 60 °C for 30 s. Gene expression was determined based on the ΔCt values between *PPM1A* and the housekeeping gene GAPDH (FAM-labelled probe, Thermo Scientific) and compared with the mean ΔCt values for the control group to determine a fold induction value.

### Real-time cell analysis assay (RTCA)

Macrophage adhesion was measured in specialized 96-well plates (E-plate 96) with the xCELLigence Real-time Cell Analyzer (RTCA) SP apparatus (ACEA Biosciences, San Diego, CA) as described previously^13^. Data was quantified by measuring impedance changes between the sensing electrodes located in the well-bottom, which changes as a function of the adhesion of cells to the surface of the plate. A dimensionless value, the Cell Index (CI), is representative of these impedance changes. Using this system, macrophage adhesion and therefore differentiation capacity was monitored in real-time. Non-adherent cells in suspension do not trigger a signal in this system. Given the sensitivity of the system, a spike in CI is observed following media exchange due to temporary changes in temperature. These spikes normally equilibrate back to stable levels within 2 h. The CI at every time point represents the mean of three independent measurements.

### Surface and intracellular staining

Cells were harvested using non-enzymatic dissociation solution (Corning, Corning, NY) and washed once with PBS. For intracellular staining of CD68, cells were treated using the Fixation/Permeabilization Solution Kit (BD Biosciences, San Jose, CA), while all other surface stains proceeded to the following step. Cells were then incubated in Human Fc Block (BD) for 10 min. Thereafter, cells were stained with FITC, PE, or APC-conjugated antibody to CD68, CD80, CD86, HLA-ABC, HLA-DR, CCR5, CD163, or CD206 in the presence of Fc Block for 30 min at 4 °C. Cells were then washed with 3× with PBS and analyzed by flow cytometry.

### Flow cytometry

Monocytes/macrophages following various stimulation or after surface/intracellular staining were analyzed by flow cytometric analysis (FCM). Corresponding isotype controls for each of the antibodies were used as controls. FCM analysis was performed on a Guava EasyCyte (Guava Technologies Inc., Billerica, MA) for THP-1 cells and a BD Accuri C6 for primary human monocytes/macrophages. Data analysis was performed using Guava Express software (Guava Technologies Inc.) or FlowJo V10 software (Ashland, OR).

### Cytokine/chemokine/phosphorylation (milliplex) assays

Macrophage supernatant or lysate following various treatments were harvested according to Milliplex kit protocol (Millipore, Billerica, MA). Protein or phosphorylation levels of various cytokines/chemokines/signaling proteins were measured using Milliplex kits. Experiments were performed according to manufacturer’s protocol and read-out was performed using a Bio-Plex 200 instrument. Data analysis was performed using Bio-Plex manager 6.0 software (Bio-Rad).

### Western blot

Cells were harvested by centrifugation, washed once with PBS, and lysed in RIPA buffer (Cell Signaling) according to the manufacturer’s instructions. Protein concentration of the lysates was determined by the bicinchoninic acid (BCA) method according to the manufacturer’s recommendations (Thermo Scientific). About 10 to 20 μg of protein per sample was separated on 10% Mini-Protean TGX gels (Bio-Rad) and subsequently transferred to a polyvinylidene difluoride (PVDF) membrane using an iBlot gel transfer system (Life Technologies). Western blot analysis was performed according to standard protocols. Total PPM1A, α-tubulin, or GAPDH proteins were detected with specific antibodies (see antibody section). A horseradish peroxidase-conjugated goat anti-rabbit or goat anti-mouse polyclonal antibody (Santa Cruz) was used as the secondary antibody. The blot was developed using the Western Lightning Ultra chemiluminescent substrate from Perkin Elmer, Inc., and detected in an EpiChemi3 darkroom (UVP BioImaging Systems).

### Statistical analysis

Data are expressed as the mean ± the standard deviation of three independent experiments. Statistical analysis was performed using the Student *t* test. Values of *p* < 0.01 were considered to be significant.

## Electronic supplementary material

Supplementary Information
