# Supplementary material for: The phosphatase PPM1A controls monocyte-to-macrophage differentiation
Source: Sci Rep. 2018 Jan 17;8:902. doi: 10.1038/s41598-017-18832-7 (PMC5772551; doi:10.1038/s41598-017-18832-7)
Supplement: Supplementary file 1 — Supplementary Information [file 41598_2017_18832_MOESM1_ESM.pdf]

SUPPLEMENTARY INFORMATION

The phosphatase PPM1A controls monocyte-to-macrophage differentiation

Samuel R Smith[2,*], Kaitlyn Schaaf[2,*], Nusrah Rajabalee[1], Frederic Wagner[2], Alexandra Duverger[2], Olaf Kutsch[2], and Jim Sun[1]

[1]Department of Biochemistry, Microbiology and Immunology, University of Ottawa, Ottawa, Ontario, Canada

[2]Department of Medicine, University of Alabama at Birmingham, Birmingham, Alabama, USA

*These authors contributed equally to this work

Correspondence to Jim Sun,

RGN 4113, 451 Smyth Road. Ottawa, Ontario, Canada K1H 8M5

Phone: 613-562-5800 ext. 8163; email: jim.sun@uottawa.ca

Running title: PPM1A controls macrophage differentiation

SUPPLEMENTARY FIGURES

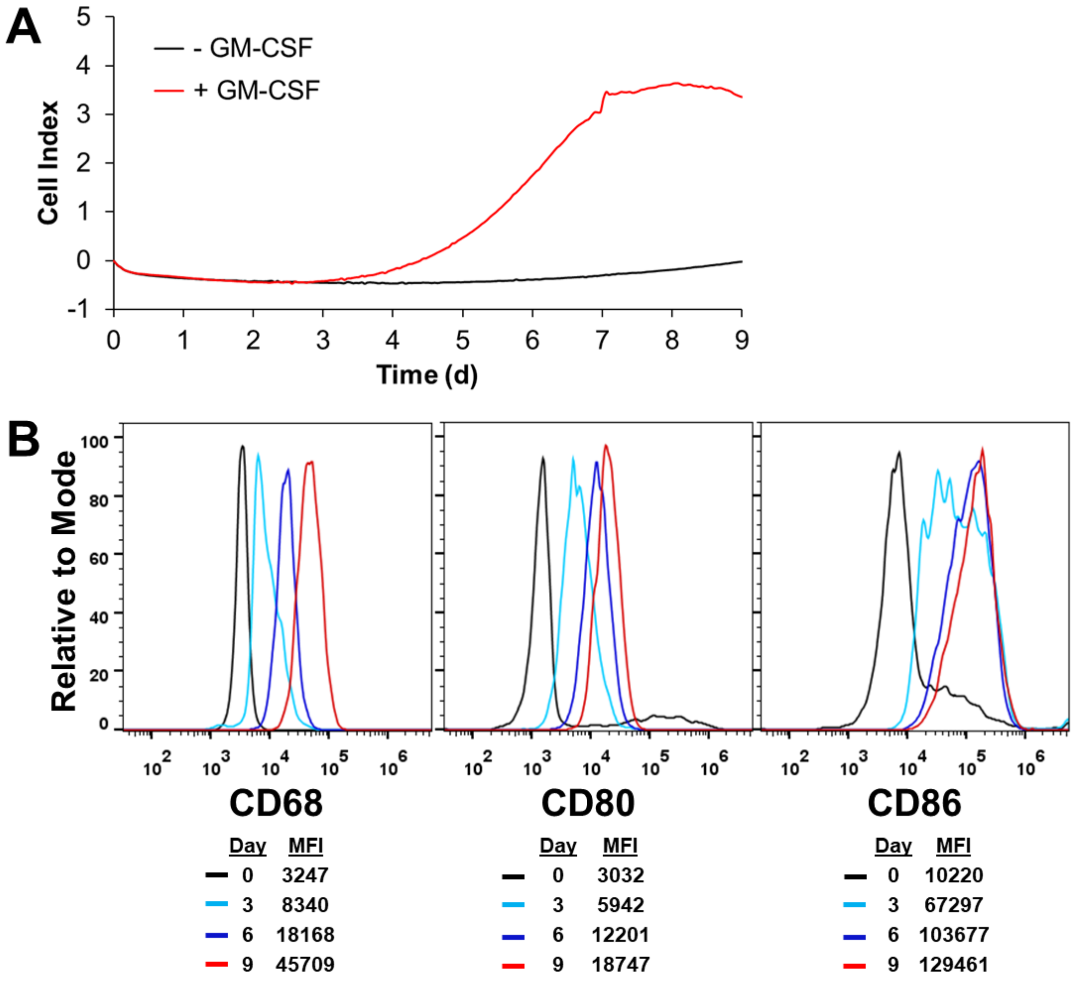

Supplementary Figure 1. Increases in expression of macrophage-specific markers is associated with a corresponding increase in Cell Index (adherence) measured by RTCA. (**A**) Primary human monocytes were left untreated or treated with GM-CSF (5 ng/ml) to induce monocyte-to-macrophage differentiation. The adherence (Cell Index) over time was measured by the RTCA instrument, and represent the average of three independent wells. (**B**) At various days post differentiation corresponding to increasing Cell Index, cells were harvested and stained for macrophage-specific markers CD68, CD80 and CD86, using FITC-conjugated antibodies. Flow cytometry was used to analyze the intensity of the marker staining and representative data is plotted here as histograms and quantified by the mean fluorescence intensity (MFI).

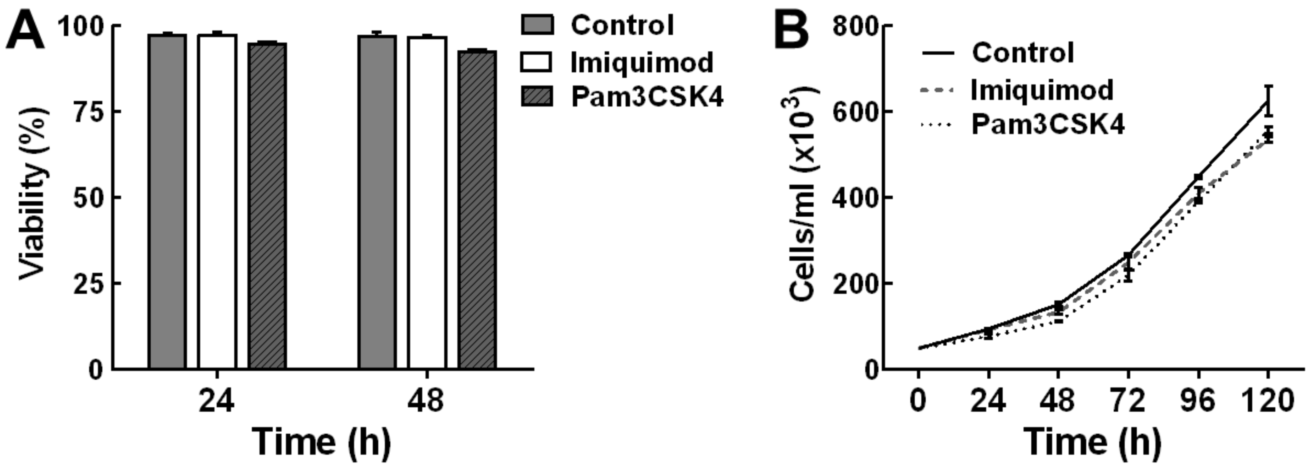

Supplementary Figure 2. Effect of imiquimod and Pam3CSK4 on monocyte viability and proliferation. (**A**) THP-1 monocytes were treated with imiquimod (5 µg/ml) or Pam3CSK4 (300 ng/ml) and viability was measured using 7-Amino Actinomycin D (7AAD) staining at 24 h and 48 h post treatment. (**B**) Cells treated as in (A) were counted by flow cytometry over a time course of 5 days to measure proliferation.

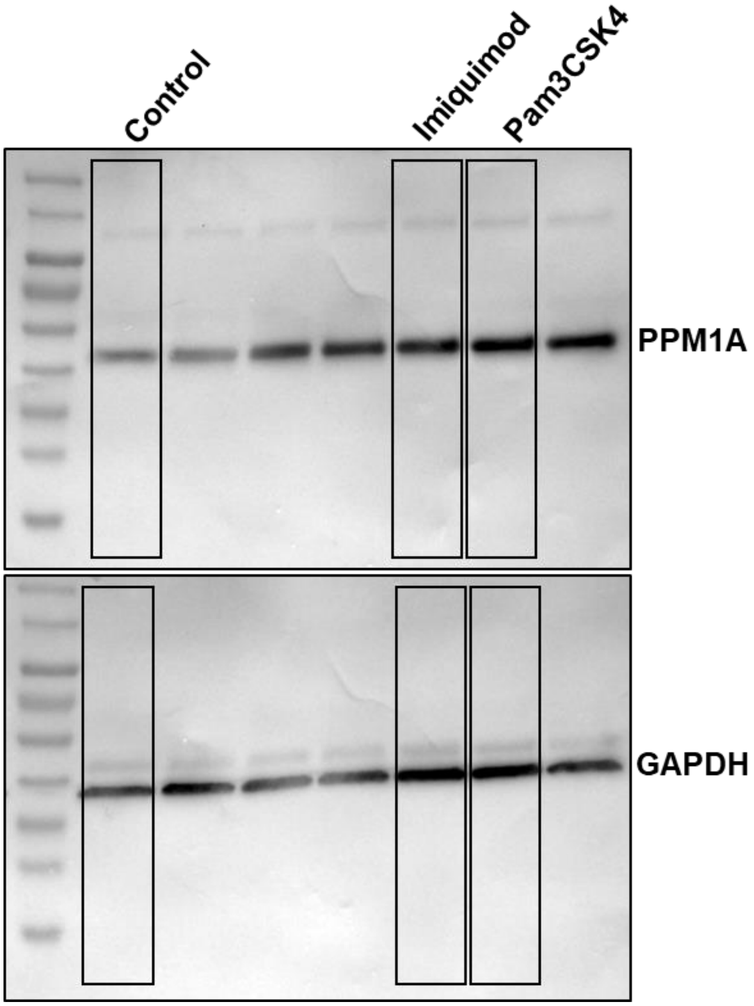

Supplementary Figure 3. TLR agonists induce upregulation of PPM1A expression: *Full-length, uncropped blot of Figure 3C*. Cell lysates from GM-CSF-differentiated hMDMs left untreated (control) or treated with imiquimod (5 µg/ml) or Pam3CSK4 (300 ng/ml) for 48 h were prepared and used to analyze PPM1A protein levels by Western blotting. Lanes corresponding to the experimental conditions are boxed. Unmarked lanes are not relevant to this experiment.

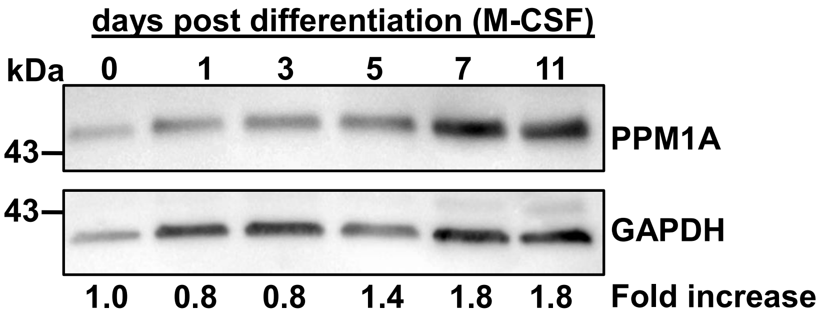

Supplementary Figure 4. PPM1A expression in response to M-CSF induced monocyte differentiation. Primary human monocytes were stimulated with M-CSF (10 ng/ml) to induce macrophage differentiation. Cell lysates were prepared at various days post differentiation and PPM1A protein levels were analyzed by Western blotting. Densitometry analysis was performed by ImageJ to quantify PPM1A band intensities as normalized to GAPDH and fold increase of PPM1A levels are expressed relative to cells prior to differentiation.

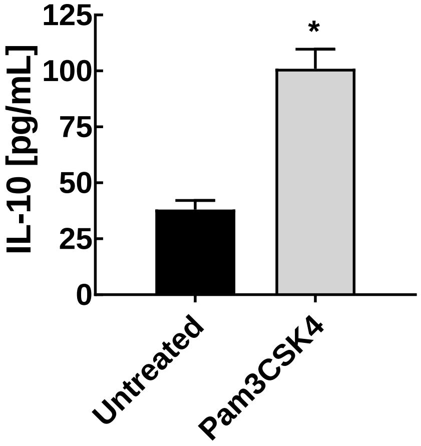

Supplementary Figure 5. PPM1A expression increases IL-10 production. Mock untreated or Pam3CSk4 (300 ng/ml) treated primary human monocytes were differentiated with GM-CSF (5 ng/ml). After 6 days of differentiation, supernatant were collected and the production of IL-10 was measured by Milliplex assays. Data represent the means ± S.D. of three independent experiments. *p < 0.01 relative to untreated cells.
